# Gut microbiota in patients after surgical treatment for colorectal cancer

**DOI:** 10.1111/1462-2920.14498

**Published:** 2018-12-19

**Authors:** Ye Jin, Yang Liu, Lei Zhao, Fuya Zhao, Jing Feng, Shengda Li, Huinan Chen, Jiayu Sun, Biqiang Zhu, Rui Geng, Yunwei Wei

**Affiliations:** ^1^ Department of Oncological and Laparoscopic Surgery The First Affiliated Hospital of Harbin Medical University Harbin China 150001

## Abstract

Colorectal cancer (CRC) is a common disease worldwide that is strongly associated with the gut microbiota. However, little is known regarding the gut microbiota after surgical treatment. 16S rRNA gene sequencing was used to evaluate differences in gut microbiota among colorectal adenoma patients, CRC patients, CRC postoperative patients and healthy controls by comparing gut microbiota diversity, overall composition and taxonomic signature abundance. The gut microbiota of CRC patients, adenoma patients and healthy controls developed in accordance with the adenoma‐carcinoma sequence, with impressive shifts in the gut microbiota before or during the development of CRC. The gut microbiota of postoperative patients and CRC patients differed significantly. Subdividing CRC postoperative patients according to the presence or absence of newly developed adenoma which based on the colonoscopy findings revealed that the gut microbiota of newly developed adenoma patients differed significantly from that of clean intestine patients and was more similar to the gut microbiota of carcinoma patients than to the gut microbiota of healthy controls. The alterations of the gut microbiota between the two groups of postoperative patients corresponded to CRC prognosis. More importantly, we used the different gut microbiota as biomarkers to distinguish postoperative patients with or without newly developed adenoma, achieving an AUC value of 0.72. These insights on the changes in the gut microbiota of CRC patients after surgical treatment may allow the use of the microbiota as non‐invasive biomarkers for the diagnosis of newly developed adenomas and to help prevent cancer recurrence in postoperative patients.

## Introduction

Colorectal cancer (CRC) is the third most common cancer and the fourth most common cause of cancer‐related death worldwide (Ferlay *et al*., 2015). Advances in cancer screening and surgical techniques have resulted in significant decreases in mortality due to CRC (Haggar and Boushey, 2009). Current estimates indicate that 20%–30% of CRC patients who undergo treatment experience recurrence and that 35% of these patients die within 5 years (Hellinger and Santiago, 2006; Ryuk *et al*., 2014; Siegel *et al*., 2017). The identification of methods to assess the risk of recurrence in patients is of tremendous importance to reduce mortality and healthcare costs.

Emerging evidence suggests that microbial dysbiosis in the human gut may be an important contributing environmental factor in CRC (Sanapareddy *et al*., 2012; Wang *et al*., 2012; Chen *et al*., 2013; Peters *et al*., 2016; Sze *et al*., 2017). Marked attention has been paid to the delineation of the gut microbiota throughout different stages of colorectal carcinogenesis (Feng *et al*., 2015; Nakatsu *et al*., 2015). Animal models, including colon tumour‐bearing mice and germ‐free mice administered stool transplants from human CRC patients, have revealed a crucial role of the gut microbiota in adenoma and CRC development (Zackular *et al*., 2013; Baxter *et al*., 2014). In addition, further steps have been taken towards the identification of non‐invasive early diagnostic biomarkers of adenoma and CRC in faecal samples (Goedert *et al*., 2015; Flemer *et al*., 2017b; Sze *et al*., 2017; Yu *et al*., 2017). These studies have suggested that gut bacteria play a positive role in tumourigenesis and that the gut likely contains high‐quality biomarkers that could potentially assist in early disease detection.

Despite advances in understanding the relationship between the gut microbiota and colorectal tumourigenesis, it remains unclear how treatments, especially tumour resection, affect the composition of the gut microbiota. It has been hypothesized that if the microbial community initiates tumourigenesis, then tumour resection should be designed to remove not only the lesion but also the gut microbiota that stimulated the tumourigenesis to reduce the risk of recurrence. To analyse this hypothesis, here we address three relevant questions: Does the resection of colorectal tumours affect the gut microbiota of CRC patients? Does tumour resection transform the gut microbiota to more closely resemble that of healthy persons or CRC patients? Furthermore, do different prognoses of postoperative patients correspond to differences in the microbial community?

To answer these questions, we designed a study to sequence the V3‐V4 region of the 16S rRNA gene amplified from faecal samples of adenoma patients, CRC patients, CRC postoperative patients and healthy controls. We characterized the gut microbiota in 116 samples and revealed that the alterations in the gut microbiota corresponded to the colorectal adenoma‐carcinoma sequence. In addition, this study further distinguished and analysed the prognoses of newly developed adenomas in CRC postoperative patients by studying different gut microbiota community compositions. The abovementioned results are expected to demonstrate that the gut microbiota can in fact be a valuable tool for the identification of biomarkers to evaluate the existence of newly developed adenomas in CRC postoperative patients.

## Results

### *Study population*

We included a total of 116 individuals in the current analysis; of these, 23 patients had tubular adenoma, 15 patients had carcinoma, 47 were CRC postoperative patients and 31 were healthy controls. For a more in‐depth analysis, the postoperative patients were further subdivided into 21 patients with newly developed adenoma (NDA) and 26 patients with a clean intestine (CIT). No significant differences in age or body mass index (BMI) were observed between any group and the healthy controls. For postoperative patients, the location of the carcinoma before surgery (proximal or distal) and the follow‐up surveillance colonoscopy time did not differ significantly. The demographic and clinical characteristics of the participants are summarized in Table 1 and Supporting Information Table S1.

**Table 1:** Demographic and clinical characteristics.

	Health control	Carcinoma	Adenoma	Postoperation		
				NDA	CIT	Total
N	31	15	23	21	26	47
Male/Female	21/10	10/5	15/8	15/6	10/16*	25/22
Age (years)	47(23–64)	63(54–83)	56.5(22–70)	58(47–74)	58.5(27–71)	58(27–74)
BMI	24.2 (16.9–38.3)	24.8 (17.3–31.4)	23.4 (16.8–33.9)	24.8 (16.8–31.2)	24.9 (16.7–31.4)	24.8 (16.7–31.4)
Resection location						
Proximal		5	8	6	10	16
Distal		10	15	15	16	31
Follow‐up (month)				22(6–36)	23.5(7–36)	22(6–36)

The Wilcoxon rank‐sum test was used to compare age and BMI between each group; Fisher's exact test was used to compare the gender distribution between the healthy controls and the other groups. Values are expressed as the median (range); *p < 0.05. BMI, body mass index; NDA, newly developed adenoma; CIT, clean intestine.

### *Global shifts in the gut microbiota of adenoma patients, carcinoma patients and healthy controls*

To investigate the alterations in the gut microbiota in patients with colorectal adenoma and carcinoma, we performed 16S rRNA gene sequencing of 69 faecal samples from healthy controls, adenoma patients and carcinoma patients. After filtering, an average of 31 802 reads per sample was obtained (range, 21 412–57 366). Using random subtraction, the sample size was equalized to 21 412 for each sample.

**Figure 1:**
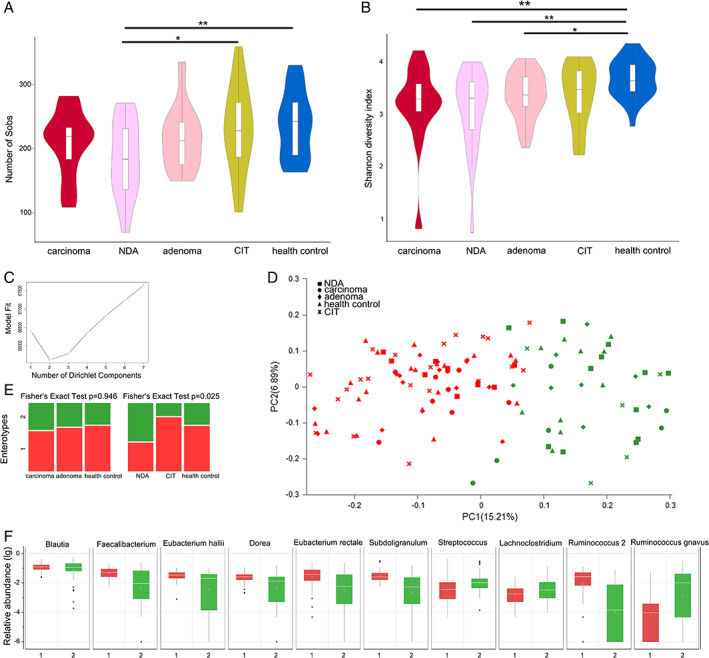
α‐Diversity and enterotypes of participants. A. The Sobs index and (B) the Shannon index of the five groups on the OTU level. Violin plots show both the richness and diversity values as well as their densities. Wilcoxon rank‐sum test, *p < 0.05, **p < 0.01. C. When the values were fit to the DMM model, the optimal classification into two community types was indicated. D. Plot of principal coordinate analysis of stool samples using DMM. Red, community type 1; green, community type 2. E. Distribution of the carcinoma, adenoma and healthy control samples and NDA, CIT and healthy control samples in the community types. The areas of the columns are scaled to the sample size. Fisher's exact test, *p* = 0.946 and *p* = 0.025. F. Relative abundances of the top 10 most abundant genera in the two community types, medians (dark lines in the boxes), the lowest and highest values within 1.5 times the interquartile range from the first and third quartiles (whiskers above and below the boxes), and outliers beyond the whiskers (circles). NDA, newly developed adenoma; CIT, clean intestine.

We first investigated the richness and evenness of the gut microbiota in the three groups. Sequencing depth was examined by plotting the rarefaction curve for richness (Supporting Information Fig. S1). Most of the samples reached plateaus, which indicates that the sequencing depth was adequate. The α‐diversities were assessed using the Sobs index and the Shannon index. The Shannon index was significantly decreased in adenoma and carcinoma patients (*p* = 0.011 and *p* = 0.005 for the adenoma and carcinoma patients respectively) compared with healthy controls (Wilcoxon rank‐sum test; Fig. 1B). However, the Sobs index was not decreased in adenoma and carcinoma patients (Fig. 1A). Thus, we could not statistically demonstrate that greater richness is a sign of a healthy gut microbiota in this cohort, although the results indicate that overgrowth of a variety of bacteria transforms the evenness of the gut microbiota in patients with colorectal adenoma and carcinoma.

Enterotype, which is another general measure of the gut microbiota, was identified in two microbial community types among the participants using a Dirichlet multinomial mixture (DMM) model (Fig. 1C). Each community type included healthy controls, adenoma patients, carcinoma patients and postoperative patients (Fig. 1D). The top 10 genera that contributed the most to the Dirichlet components are shown in Fig. 1F. In contrast to carcinoma patients, a large percentage of adenoma patients (15/23) were observed to have the same enterotype as healthy controls (Fig. 1E). The α‐diversities and enterotypes both revealed shifts in the gut microbiota during the development of CRC.

### *Microbiota development according to the adenoma‐carcinoma sequence*

**Figure 2:**
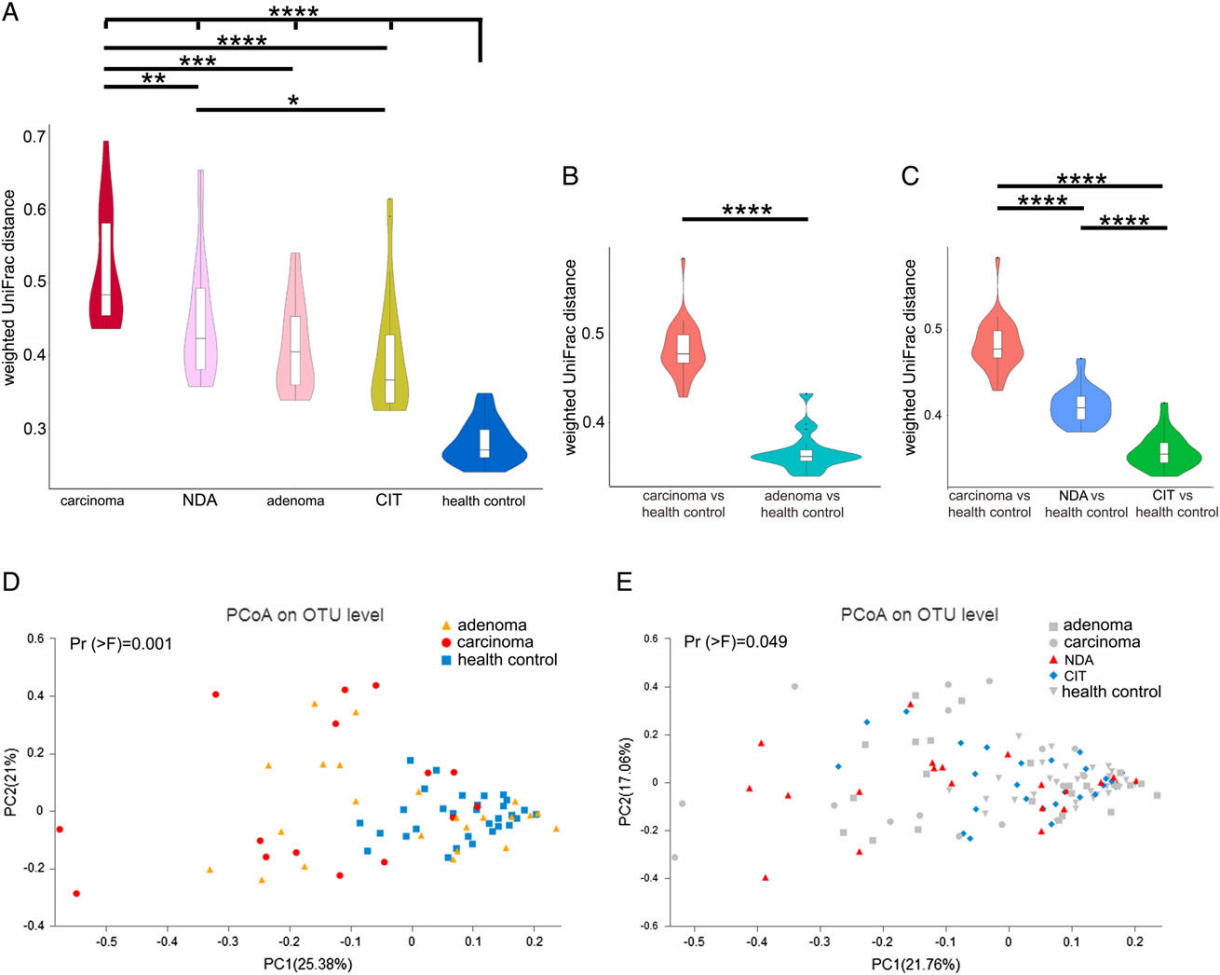
β‐diversity within and between groups. β‐diversity is represented by weighted UniFrac distances between gut microbial communities (A) and among the five groups (B, C). The box plot illustration is provided in Fig. 1. D. Principal coordinate analysis of the weighted UniFrac distance in the healthy controls, adenoma patients, carcinoma patients and (E) in the five groups. Wilcoxon rank‐sum test, *p < 0.05, **p < 0.01, *** *p* < 0.001, **** *p* < 0.0001. NDA, newly developed adenoma; CIT, clean intestine.

β‐Diversity, which is represented by principal coordinates analysis (PCoA), is based on the weighted UniFrac and showed that the bacterial composition of carcinoma patients was clearly segregated from that of healthy controls (permutational multivariate analysis (PERMANOVA) test, Pr (>F) = 0.001); the same result was found when adenoma patients and healthy controls were compared (PERMANOVA test, Pr (>F) = 0.033; Fig. 2D). Then, we tested the inner‐group and outer‐group distances in the three groups. This analysis showed that the inner‐group difference was more significant in carcinoma patients than in healthy controls (Wilcoxon rank‐sum test, *p* < 0.0001; Fig. 2A). Moreover, a smaller inner‐group difference was observed in adenoma patients than in carcinoma patients (Wilcoxon rank‐sum test, *p* = 0.0001 Fig. 2A). The distance between carcinoma patients and healthy controls was larger than that between adenoma patients and healthy controls (Wilcoxon rank‐sum test, *p* < 0.0001 Fig. 2B). These results revealed that the bacterial composition of adenoma patients was more similar to that of healthy controls than to that of carcinoma patients according to the weighted UniFrac distances. This finding was supported by the comparison of the taxonomic distribution of all three groups at the phylum, family and genus levels (Supporting Information Fig. S2).

**Figure 3:**
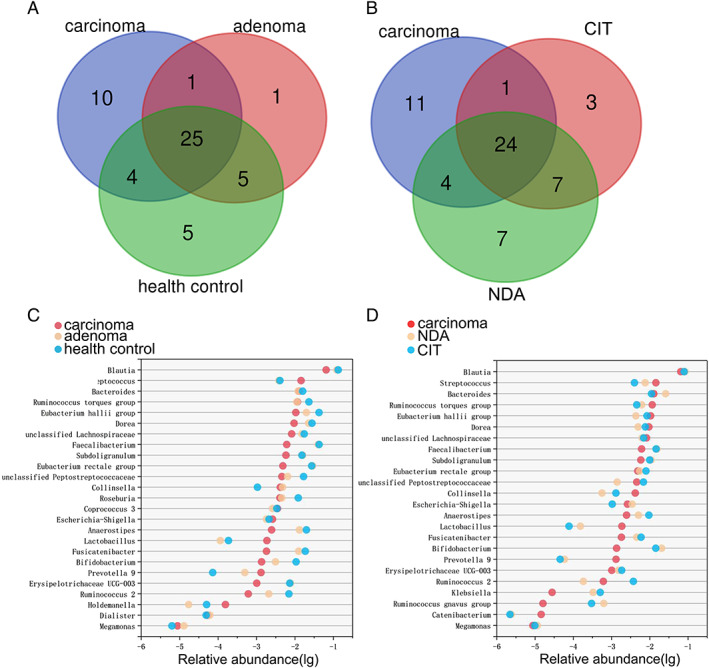
Genera with a respective relative abundances above 0.5% in the samples from healthy controls and from patients with adenoma, carcinoma, NDA or CIT. A and B. Venn diagram showing the common genera between groups. C and D. Relative abundance of the common genera in each group. NDA, newly developed adenoma; CIT, clean intestine.

The genera with average abundance levels >0.5% in adenoma patients, carcinoma patients and healthy controls were used to construct Venn diagrams, which are commonly used to display gut microbiota overlap between groups. The Venn diagrams showed large overlap between the adenoma patients and healthy controls, as well as that between adenoma patients and CRC patients (Fig. 3A). However, there were few different bacteria between CRC patients and healthy controls. Among the genera that were shared among the three groups were populations generally regarded as beneficial to the host (e.g., *Faecalibacterium, Bacteroides* and *Dorea*). The relative abundance of beneficial gut microbiota in the three groups exhibited gradient changes (Fig. 3C). These analyses revealed impressive shifts in the gut microbiota before or during the development of CRC that developed in accordance with the adenoma‐carcinoma sequence.

### *Notable differences in the gut microbiota between CRC and postoperative patients*

We also evaluated the gut microbiota of patients who underwent surgical resection of CRC to investigate if the treatment altered the microbial community. Regarding α‐diversity, the Sobs index and the Shannon index of the postoperative patients more closely resembled those of the CRC patients than those of the healthy controls (Supporting Information Fig. S3). With respect to β‐diversity, the PCoA based on the weighted UniFrac showed that the composition of the gut microbiota differed between postoperative patients and CRC patients (PERMANOVA test, Pr (>F) = 0.005, Supporting Information Fig. S4). In the Venn diagram, the gut microbiota of the CRC and postoperative patients showed marked overlap at the genus level, with more genera observed in patients who underwent surgery than in those who did not undergo surgery (Supporting Information Fig. S5A). Comparison of the taxonomic distributions of the gut microbiota of CRC and postoperative patients showed marked alterations at the phylum, family and genus levels (Supporting Information Fig. S6). In terms of specific bacteria, 34 genera differed between postoperative patients and CRC patients (Wilcoxon rank‐sum test, *p* < 0.05, Supporting Information Fig. S5B, Table S2). Multiple hypothesis tests revealed that six differences in bacterial composition at the genus level, including *Gemella*, *Tyzzerella 3*, unclassified *Oxalobacteraceae*, *Howardella*, *Lawsonella* and *Parascardovia* (*Q* < 0.1). These results showed that the gut microbiota was clearly different between CRC and postoperative patients.

### *The gut microbiota of NDA patients is similar to that of carcinoma patients*

For further study, we divided the postoperative patients into two groups according to their colonoscopy findings: the NDA group and the CIT group. The Sobs index was significantly reduced in the NDA group compared with the healthy control group (Wilcoxon rank‐sum test, *p* = 0.005) and the CIT group (Wilcoxon rank‐sum test, *p* = 0.02; Fig. 1A). However, similar differences were not observed between the other groups. Similarly, the Shannon index was also significantly decreased in the NDA group compared with the healthy control group (Wilcoxon rank‐sum test, *p* = 0.003; Fig. 1B), whereas no difference was observed between the CIT group and the healthy control group.

PCoA revealed differences in bacterial composition between the NDA and CIT patients based on the weighted UniFrac (PERMANOVA test, Pr (>F) = 0.049; Fig. 2E), and NDA patients had larger inner‐group distances than CIT patients (Wilcoxon rank‐sum test, *p* = 0.023; Fig. 2A). The distance between NDA patients and healthy controls was larger than that between CIT patients and healthy controls (Wilcoxon rank‐sum test, *p* < 0.0001) but was smaller than the distance between carcinoma patients and healthy controls (Wilcoxon rank‐sum test, *p* < 0.0001; Fig. 2C). In addition, for the composition of gut microbiota, greater overlap was also observed in the Venn diagram between NDA patients and carcinoma patients than between CIT patients and CRC patients (Fig. 3B). These results revealed the gut microbiota of NDA patients was more similar to that of CRC patients, while the gut microbiota of CIT patients was more similar to that of the healthy controls.

**Figure 4:**
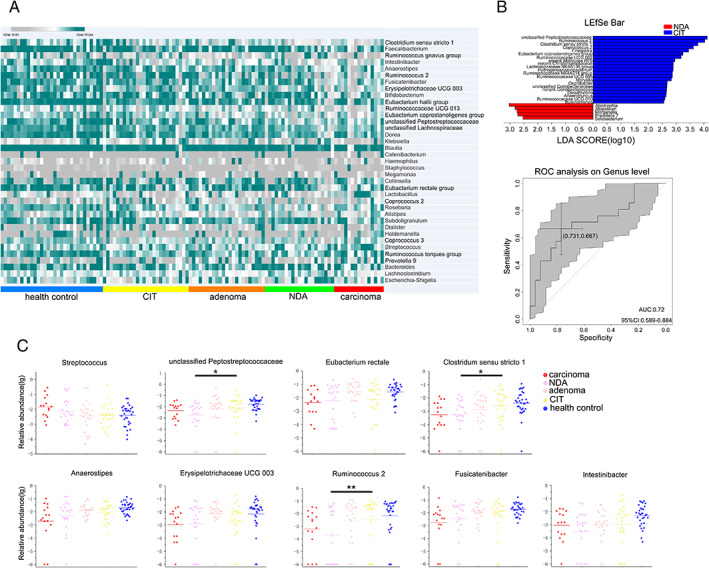
Characteristics of taxonomic signatures. A. Colour‐coded heat map displaying the amounts of related metabolites in the five groups. The colour scale represents the scaled abundance of each genera with relative abundances above 0.5% from the five groups, which was denoted as the *Z*‐score; green and grey indicate increased and decreased abundance respectively. B. LEfSe identified the taxa with the greatest differences in abundance between NDA and CIT. At the genus level, taxa enriched in CIT patients are indicated by a positive LDA score (blue), and NDA patient‐enriched taxa are indicated by a negative score (red). Only taxa meeting a significant LDA threshold value of >2.5 are shown. Receiving operating characteristic curve analysis was used to assess the predictive model performance between NDA and CIT. C. The percentage and relative abundance of nine types of bacteria in the five groups. Wilcoxon rank‐sum test, *p < 0.05, **p < 0.01. NDA, newly developed adenoma; CIT, clean intestine.

We then explored the taxonomic signatures at the genus level. Genera with average abundance levels > 0.5% (37 genera) in the five groups were used to construct the heatmap. The upper half of the heatmap showed an alteration in the concentration gradient from healthy controls to carcinoma patients that corresponded to the adenoma‐carcinoma sequence (Fig. 4A; Supporting Information Table S3). Fifteen of the 37 genera were observed to have significant differences in abundance between carcinoma patients and healthy controls (Wilcoxon rank‐sum test, *p* < 0.05, false discovery rat (FDR), *Q* < 0.1). Carcinoma patients exhibited a greater abundance of *Streptococcus*, *Lactobacillus* and *Prevotella 9* and a decreased abundance of *Faecalibacterium*, *Dorea* and *Ruminococcus 2* compared with healthy controls (Supporting Information Table S4). Nine of the genera were *Streptococcus*, *Ruminococcus 2, Fusicatenibacter, Anaerostipes, Erysipelotrichaceae UCG‐003, Intestinibacter, Clostridium sensu stricto 1, Escherichia‐Shigella, Eubacterium rectale group* and unclassified *Peptostreptococcaceae* were observed in line with the adenoma‐carcinoma sequence for postoperative patients (Fig. 4C).

Finally, we attempted to determine the correlation between the clinical monitoring index of CRC and the gut microbiota in postoperative patients. Carcinoembryonic antigen (CEA) and carbohydrate antigen 19‐9 (CA19‐9), which are the most common clinical tumour markers for CRC, were used to correlate the clinical index with the gut microbiota at the genus level by Spearman correlation analysis. *Atopobium* was observed to be positively correlated with CEA, while *Butyricimonas, Sellimonas, Turicibacter* and an unclassified genus of *Lactobacillales* were negatively correlated with CEA. A correlation was also observed between CA19‐9 and *Eisenbergiella*, *Prevotella 2* and an unclassified genus of *Erysipelotrichaceae* (Supporting Information Fig. S7 and Table S5). However, the levels of CEA and CA19‐9 in the NDA group were not significantly different from those in the CIT group (Wilcoxon rank‐sum test). This might be because CEA and CA19‐9 are biomarkers for CRC, whereas adenoma is a precancerous lesion. Therefore, the levels of CEA and CA19‐9 in the NDA group were not as high as those in the CRC group. Although we did not observe significant differences in the levels of CEA and CA19‐9 between the NDA and CIT patients, our results showed a notable difference in the gut microbiota between these two groups. Despite the low levels of CEA and CA19‐9, we also found a correlation between these two markers and the gut microbiota of the NDA and CIT groups. To assess the degree of differentiation of the gut microbiota in the NDA and CIT groups, a linear discriminant analysis effect size (LEfSe) analysis revealed significant differences (linear discriminant analysis (LDA >2.5; *p* < 0.05) between the two groups in 26 genera. We then attempted to determine if these differences in the gut microbiota could serve as potential biomarkers for noninvasive monitoring and diagnosis of NDA in postoperative patients. We applied random forest, which can avoid model overfitting, to build distribution model. The performance of the model was assessed using receiver operating characteristic (ROC) analysis. Finally, the first 10 species based on the LDA value were selected from the 26 different genera as biomarkers, achieving an area under curve (AUC) value of 0.72 (Fig. 4B, Supporting Information Table S6). These analyses revealed that the gut microbiota may be related to these surveillance markers of recurrent CRC and that gut microbiota may be used as non‐invasive biomarkers for the diagnosis of NDA to prevent cancer recurrence in postoperative patients.

## Discussion

In this study, we compared the gut microbiota of adenoma patients, carcinoma patients and postoperative patients with those of patients with NDA or CIT and healthy controls. We observed changes in the gut microbiota among all groups, especially in NDA and CIT patients. We also confirmed that the variations of gut microbiota among adenoma patients, carcinoma patients and healthy controls corresponded to the progression of the disease and that the gut microbiota of NDA patients was more comparable to the gut microbiota of carcinoma patients than to the gut microbiota of healthy controls. This study is a valuable investigation of alterations in the gut microbiota of postoperative patients with CRC and the first to assess the gut microbiota of postoperative patients with or without NDA.

First, we analysed and compared the global gut microbiota properties of adenoma patients, carcinoma patients and healthy controls. Our finding of reduced species richness and diversity in adenoma patients, particularly in carcinoma patients, differed from the findings of some previous studies conducted in France and Austria. Those studies showed that CRC‐associated dysbiosis did not result in significant changes in microbial community diversity or richness (Zeller *et al*., 2014; Feng *et al*., 2015). Although this difference in outcome may be ascribed to different populations and dietary habits, our findings are in agreement with those of other reports (Yu *et al*., 2017). Notably, the gut microbiota differed significantly between CRC patients and postoperative patients. Furthermore, more obvious differences in gut microbiota were found when NDA and CIT patients were compared. Our result may suggest that surgery for CRC is not only successful in removing the tumour but also in altering the associated bacterial communities.

Next, we showed that the development of CRC was accompanied by changes in the gut microbiota, as evidenced by the comparison of healthy controls, adenoma patients and carcinoma patients. It is generally accepted that most CRCs develop according to a continuous process in which the normal mucosa is transformed into adenoma and then to carcinoma (Muto *et al*., 1975; Stryker *et al*., 1987; Weitz *et al*., 2005), which is termed the adenoma‐carcinoma sequence (Fearon *et al*., 1987; Vogelstein *et al*., 1988; Brenner *et al*., 2014). Our results showed that with the progression of CRC, the gut microbiota also exhibited stages of alterations. Feng and colleagues demonstrated the development of gut microbiota along the colorectal adenoma‐carcinoma sequence by shotgun sequencing of faecal samples, and Nakatsu and colleagues obtained similar results for mucosal microbiota (Feng *et al*., 2015; Nakatsu *et al*., 2015). Based on these data, we enrolled postoperative patients in this study to investigate whether the gut microbiota is altered after colon surgery. We observed obvious differences between carcinoma and postoperative patients. More significant differences were found when we divided postoperative patients into those with NDA, a potential risk for cancer, and those with CIT.

Furthermore, we were surprised to observe not only a significant difference in the gut microbiota between NDA and CIT patients but also that the microbial community of NDA and CIT patients was representative of the respective disease state. In other words, our results showed that the gut microbiota of NDA and CIT patients corresponded with the adenoma‐carcinoma sequence. Thus, the different alterations in the gut microbiota after surgical resection of CRC may influence the prognosis of the disease.

Finally, we investigated the taxonomic signature of the five groups at the genus level. Emerging evidence has indicated that microbial dysbiosis may be an important factor for CRC (Flemer *et al*., 2017a; Yu *et al*., 2017; Shah *et al*., 2018). Tsoi and colleagues reported that *Peptostreptococcus* induces cell proliferation and dysplasia in mice (Tsoi *et al*., 2017). Many studies have suggested that in humans, *Bacteroides* and *Escherichia* may promote colorectal carcinogenesis (Cuevas‐Ramos *et al*., 2010; Arthur *et al*., 2012; Grivennikov *et al*., 2012), and other studies have revealed associations between CRC and clinical infections by particular bacteria such as *Streptococcus* (Boleij *et al*., 2009). In our study, nine genera were observed to be significantly different between CRC patients and healthy controls, which also corresponded to the adenoma‐carcinoma sequence. The relative abundance of *Streptococcus* was increased in NDA patients compared with CIT patients, although this difference was not statistically significant. This result may have been obtained because NDA is considered a precancerous lesion, and therefore, the alterations in the gut microbiota were less evident than those in CRC. However, among the nine genera, *Ruminococcus 2*, *Clostridium sensu stricto 1* and an unclassified *Peptostreptococcaceae* genus had significantly increased abundances in CIT patients compared with NDA patients. Many reports have been published about the prospect of the application of the gut microbiota as a non‐invasive biomarker for the diagnosis of CRC (Zeller *et al*., 2014; Liang *et al*., 2017; Yu *et al*., 2017). In our study, we used the top 10 species based on the LDA value to distinguish NDA and CIT, achieving an AUC value of 0.72. Based on the results of this study, we speculate that it may be possible to use the gut microbiota as a biomarker to assess the presence of NDA in postoperative patients.

Two studies have been published on the gut microbiota in postoperative patients who underwent surgery for CRC. Seiji Ohigashi and colleagues reported significant changes in the intestinal environment and short‐chain fatty acids (Ohigashi *et al*., 2013). However, they did not use next‐generation sequencing to investigate the alterations in the gut microbiota, and their faecal samples were collected on the seventh day after surgery. Sze and colleagues used a random forest machine learning algorithm to classify pre‐ and post‐treatment CRC patients, and the gut microbiota they identified could possibly be used to quantify the risk of recurrence (Sze *et al*., 2017). In contrast to these two studies, we investigated the alterations in the gut microbiota of postoperative patients with NDA or CIT; our study also featured the longest follow‐up time (median, 22 months). However, several limitations must be addressed in future studies. First, this was a relatively small study with limited sample size, and the diagnostic potential of the selected biomarkers should be evaluated in an independent cohort. Therefore, further studies are needed with larger samples to eventually validate the predictive power of the selected biomarkers. Second, the gut microbiota analysis was performed on faecal samples but not on tissue samples. Although tissue samples represent the local microbiota and might be more relevant to the development of adenoma in CRC postoperative patients and the identification of novel biomarkers for the diagnosis of NDA in postoperative patients in real time, faecal samples are more freely available than tissues. Tissue samples of CRC postoperative patients must be obtained through colonoscopy, which is an invasive procedure and cannot be tolerated in some patients. In addition, in CRC postoperative patients with CIT, collecting intestinal tissue samples may cause additional damage (for example, bleeding). Therefore, we chose to analyse the gut microbiota from faecal samples in the current research. Finally, the design of our study was cross‐sectional; thus, we did not collect preoperative and postoperative faecal samples from the same CRC patients. However, comparing the gut microbiota between preoperative patients and postoperative recurrence patients may provide guidance for postoperative treatment, including chemotherapy, radiotherapy and probiotics. Regarding this aspect, future before‐and‐after studies of gut microbiota from CRC patients are needed.

Despite these limitations, we observed obvious differences in gut microbiota between carcinoma and postoperative patients as well as a significant difference between NDA and CIT patients. Furthermore, the alterations in the gut microbiota between the NDA and CIT patients corresponded to disease progression. We believe that the differences in the gut microbiota between NDA and CIT patients may serve as novel biomarkers for the diagnosis of NDA to prevent cancer recurrence in postoperative patients.

## Experimental procedures

### *Study population*

Recruitment and sampling of participants was carried out at the First Affiliated Hospital of Harbin Medical University from May 2016 to December 2017. Eligible participants were individuals at least 18 years of age and younger than 85 who recently underwent colonoscopy, were able to communicate and make decisions, and who had not received long‐term antibiotic treatment. The adenoma and carcinoma patients were diagnosed by colonoscopic examination and histopathological review of biopsies. Postoperative patients were classified as having NDA or CIT by follow‐up surveillance colonoscopy and biopsies. Healthy controls who were recruited from the health screening centre were required to have a clear colonoscopy and were matched for age, gender and BMI. All samples were collected before colonoscopy or 2 months after colonoscopy.

Follow‐up samples of 47 individuals were obtained between six and 36 months after surgical resection. The exclusion criteria included the following: patients with permanent ostomy, distant metastasis, chronic renal disease or hepatic cirrhosis, chronic ischaemic heart disease with unstable angina, chronic heart failure of class III or IV or acute myocardial infarction in the last 6 months; those with a history of chronic diarrhoea, a history of diabetes mellitus or a history of autoimmune diseases; those who used antibiotics or probiotics 3 months before sample collection; those with a history of other abdominal surgery for any reason; and those with any history of cancer other than CRC, inflammatory bowel disease and any known disease that may influence the gut microbiota.

The clinical trial was approved by the Research Ethics Committee of the First Affiliated Hospital of Harbin Medical University. The studies were strictly performed according to international guidelines regarding the conduct of clinical trials, and each patient provided written informed consent. This study was registered at ClinicalTrials.gov and began on May 1, 2016 (NCT03385213).

### *Sampling, DNA extraction and PCR amplification*

Each participant provided a fresh stool sample in the hospital, and the sample was delivered immediately to the laboratory in an insulated box. Upon collection, the faecal sample was immediately divided into aliquots that were then frozen in liquid nitrogen immediately and stored at −80°C until further analysis. Microbial DNA was extracted from the faeces using an E.Z.N.A.® stool DNA Kit (Omega Bio‐tek, Norcross, GA, USA) according to the manufacturer's instructions. The V3‐V4 hypervariable regions of the bacterial 16S rRNA gene were amplified in a thermocycler PCR system (GeneAmp 9700, ABI, MA, USA) using the following primer pairs: forward 338‐CCTAYGGGRBGCASCAG and reverse 806‐GGACTACNNGGGTATCTAAT.

### *16S rRNA gene sequencing*

Purified amplicons were pooled in equimolar concentrations and sequenced on an Illumina MiSeq platform (Illumina, San Diego, CA, USA) in PE300 mode according to the standard protocols provided by Majorbio Bio‐Pharm Technology (Shanghai, China). Raw FASTQ files were demultiplexed, quality‐filtered by Trimmomatic and merged by FLASH according to the following criteria. (i) The reads were truncated at any site and received an average quality score < 20 over a 50 bp sliding window; (ii) primers were matched exactly, allowing two‐nucleotide mismatching, and reads containing ambiguous bases were removed; (iii) sequences with overlaps longer than 10 bp were merged according to their overlapping sequence.

### *Preliminary data processing and quality control*

16S rRNA gene sequencing data were processed using the Quantitative Insights Into Microbial Ecology platform (QIIME; V.1.9.1). Operational taxonomic units (OTUs) were selected according to a cut‐off of 97% similarity, and the identified taxonomy was then aligned using the Greengenes database (V.13.8). Chimeric sequences were identified and deleted. A rarefaction curve was constructed using the Sobs and Shannon index to prevent methodological artefacts originating from variations in sequencing depth.

### *Bioinformatics analysis*

The raw counts of 221 501 de novo OTUs were agglomerated to 19 phyla, 30 classes, 54 orders, 92 families, 279 genera and 943 OTUs. We then isolated low‐count taxa with richness greater than 0.5% in the OTU sequence reads in each group, which were then used in the Venn and taxonomic signature analyses (described below).

Within‐subject microbial diversity (α‐diversity) was assessed using species richness (Sobs) and the Shannon diversity index, which were calculated in 1000 iterations of rarefied OTU tables at 21 412 sequence reads per sample. This sequencing depth was chosen to sufficiently reflect the diversity of the samples while retaining the maximum number of participants for the analysis.

β‐Diversity was estimated by computing the weighted UniFrac distance and was visualized using PCoA; the results were plotted using the WGCNA, stats and ggplot2 packages in R software (Version 2.15.3).

PERMANOVA of the distance matrices, as implemented in the ‘vegan’ package in R, was used to identify whether case/control status explained variation in microbial community composition.

The stool samples were classified into community types or enterotype based on whether they possessed a similar microbial composition at the genus level. This was achieved with the use of a DMM model (Holmes *et al*., 2012), which was implemented using the ‘Dirichlet Multinomial’ package in R. The top 10 phylotypes that contributed to this model were visualized.

The random forest algorithm using R package was used to create the models used to classify NDA and CIT samples. To evaluate the discriminatory ability of the prediction model, ROC were constructed, and AUC values were calculated. The first 10 species based on the LDA value were selected from the 26 different genera as biomarkers, and set NDA and CIT as 0 and 1, using R package(pROC) to draw the ROC curve and achieving an AUC value. This function computes the confidence interval of the specificity at the given sensitivity points. By default, the 95% CI are computed with 2000 stratified bootstrap replicate.

### *Statistical methods*

All statistical analyses were performed using the R package and SPSS 19.0 software. The microbiota features differentiating the faecal microbiota were characterized using the LEfSe method for biomarker discovery, which emphasizes both statistical significance and biological relevance. Based on a normalized relative abundance matrix, LEfSe uses the Kruskal–Wallis rank‐sum test to detect features with significantly different abundance levels between assigned taxa and performs an LDA to estimate the effect size of each feature. Correlations between variables were computed using the Spearman rank correlation. All tests for significance were two‐sided, and *p* values < 0.05 were considered significant. Multiple hypothesis tests were adjusted using the Benjamini and Hochberg FDR, and differences were considered significant when the results were below an FDR threshold of 0.1. Only bacterial taxa with average abundances >0.5% were compared between groups in the analysis of significant difference using the Wilcoxon rank‐sum test.

## Ethics approval and consent to participate

The clinical trial was approved by the Research Ethics Committee of the First Affiliated Hospital of Harbin Medical University (IRB‐AF/SC‐08/05.0). The studies were strictly performed according to international guidelines regarding the conduct of clinical trials, and each patient provided written informed consent.

## Author contributions

Yunwei Wei and Ye Jin made substantial contributions from conception of the experiment to the design and acquisition of data. Ye Jin, Yang Liu and Shengda Li collected samples. Wei Yunwei, Fuya Zhao, Jing Feng and Zhao Lei carried out clinical diagnosis and treatment. Liu Yang, Huinan Chen, Jiayu Sun and Biqiang Zhu performed bioinformatics and statistical analyses and interpreted the data. Yunwei Wei, Ye Jin and Rui Geng wrote the paper. All authors read and approved the final manuscript.

## Supporting information


**Fig. S1.** Rarefaction curves exhibited the OTU richness and evenness in healthy control, adenoma patients, carcinoma patients and postoperative patients with newly developed adenoma (NDA) or clean intestine (CIT).
**Fig. S2.** The relative abundance of faecal bacterial phyla, family and genus were clustered into each groups. All OTUs with lower abundances were grouped as ‘others’.
**Fig. S3.** Richness and a‐diversity (Shannon index) of the OTU level from healthy control, adenoma, carcinoma and postoperative patients.
**Fig. S4.** Principal‐coordinated analysis based on Weighted Unifrac of healthy control, adenoma, carcinoma and postoperative patients.
**Fig. S5.** The difference of microbiota between carcinoma patients and postoperative patients in genus level. (A) Venn diagram showing the common genus between groups. (B) The relative abundance of genus in two groups. Wilcoxon rank‐sum test, *p < 0.05, **p < 0.01.
**Fig. S6.** The relative abundance of faecal bacterial phyla, family and genus were clustered between carcinoma patients and postoperative patients. All OTUs with lower abundances were grouped as ‘others’.
**Fig. S7.** Colour‐coded heatmap displaying the relationship among microbiota, CEA, CA19‐9, age and BMI. The colour scale represents the correlation coefficient of bacteria and clinical index, with red and green indicating a positive and negative correlation respectively. *p < 0.05, **p < 0.01. BMI, body mass index; CA19‐9, carbohydrate antigen 19–9; CEA, carcino embryonic antigen.

**Fig. S8.** Receiver operating characteristic of selected genus

**Table S1.** Clinical information for all samples.

**Table S2.** The relative abundance of microbiota in genus level between carcinoma patients and postoperative patients.

**Table S3.** The average relative abundance of microbiota for five groups in heatmap.

**Table S4.** The statistical difference abundance of genera with average abundance levels >0.5% between carcinoma patients and healthy controls.

**Table S5.** The relationship among microbiota, CEA, CA19‐9, age and BMI.

**Table S6.** The LEfSe result of NDA and CIT in postoperative patients.

**Table S7.** OUT table for all samples.

## Data Availability

The raw sequences have been deposited in the NCBI Sequence Read Archive (SRP144012), and the necessary metadata can be found at https://www.ncbi.nlm.nih.gov/Traces/study/ by searching the respective SRA study accession. This study was registered at ClinicalTrials.gov and began on May 1, 2016 (NCT03385213).
