# Supplementary material for: Gut microbiota in patients after surgical treatment for colorectal cancer
Source: Environ Microbiol. 2018 Dec 19;21(2):772–83. doi: 10.1111/1462-2920.14498 (PMC7379540; doi:10.1111/1462-2920.14498)
Supplement: Supplementary file 1 — Fig. S1. Rarefaction curves exhibited the OTU richness and evenness in healthy control, adenoma patients, carcinoma patients and postoperative patients with newly developed adenoma (NDA) or clean intestine (CIT). Fig. S2. The relative abundance of faecal bacterial phyla, family and genus were clustered into each groups. All OTUs with lower abundances were grouped as ‘others’. Fig. S3. Richness and a‐diversity (Shannon index) of the OTU level from healthy control, adenoma, carcinoma and postoperative patients. Fig. S4. Principal‐coordinated analysis based on Weighted Unifrac of healthy control, adenoma, carcinoma and postoperative patients. Fig. S5. The difference of microbiota between carcinoma patients and postoperative patients in genus level. (A) Venn diagram showing the common genus between groups. (B) The relative abundance of genus in two groups. Wilcoxon rank‐sum test, *p < 0.05, **p < 0.01. Fig. S6. The relative abundance of faecal bacterial phyla, family and genus were clustered between carcinoma patients and postoperative patients. All OTUs with lower abundances were grouped as ‘others’. Fig. S7. Colour‐coded heatmap displaying the relationship among microbiota, CEA, CA19‐9, age and BMI. The colour scale represents the correlation coefficient of bacteria and clinical index, with red and green indicating a positive and negative correlation respectively. *p < 0.05, **p < 0.01. BMI, body mass index; CA19‐9, carbohydrate antigen 19–9; CEA, carcino embryonic antigen. [file EMI-21-772-s008.docx]

**Gut microbiota in patients after surgical treatment for colorectal cancer**

**Ye Jin^1, *^, Yang Liu^1, *^, Lei Zhao^1^, Fuya Zhao^1^, Jing Feng^1^, Shengda Li^1^, Huinan chen^1^, Jiayu Sun^1^, Biqiang Zhu^1^, Rui Geng^1^, Yunwei Wei^1, +^**

^1^Department of Oncological and Laparoscopic Surgery, The First Affiliated Hospital of Harbin Medical University, Harbin, Heilongjiang 150001, People’s Republic of China

**^+^**Correspondence and requests for materials should be addressed to Yunwei Wei, email: [hydwyw11@hotmail.com](mailto:hydwyw11@hotmail.com);

**^*^**these authors contributed equally to this work


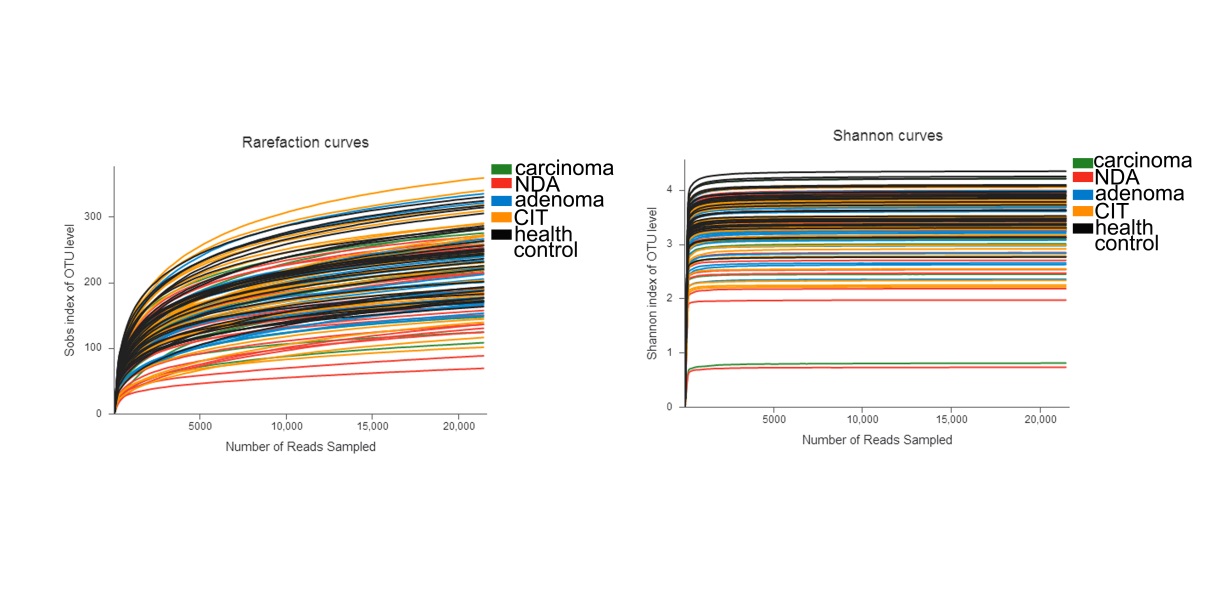
**Figure S1.** Rarefaction curves exhibited the OTU richness and evenness in healthy control, [adenoma](javascript:void(0);) patients, carcinoma patients and postoperative patients with newly developed adenoma (NDA) or clean intestine (CIT).


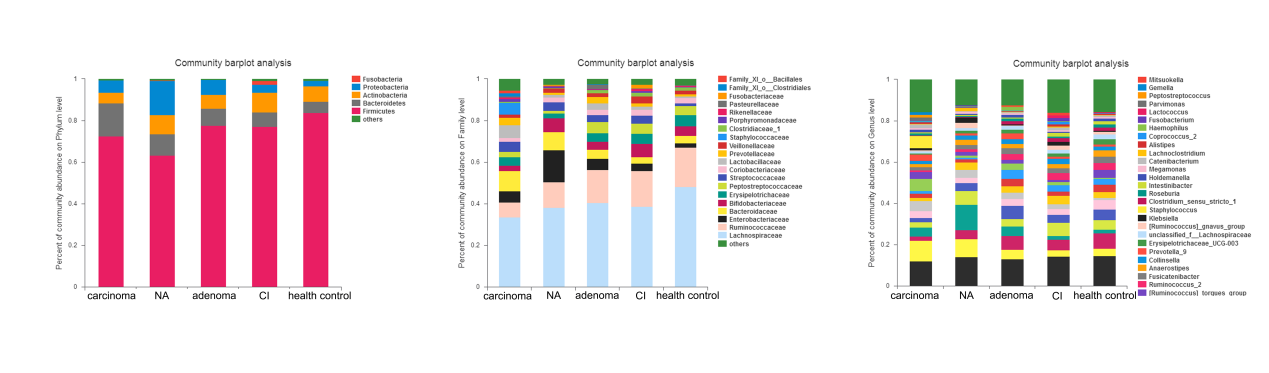


**Figure S2.** The relative abundance of faecal bacterial phyla, family and genus were clustered into each groups. All OTUs with lower abundances were grouped as “others”.


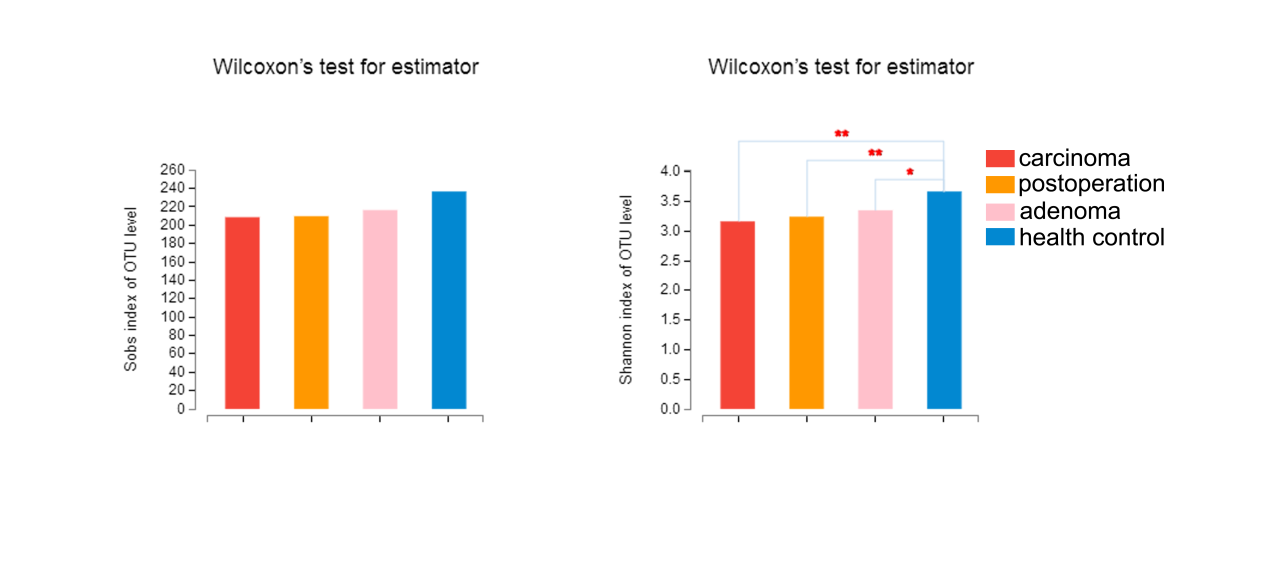


**Figure S3.** Richness and a-diversity (Shannon index) of the OTU level from healthy control, [adenoma](javascript:void(0);), carcinoma and postoperative patients.


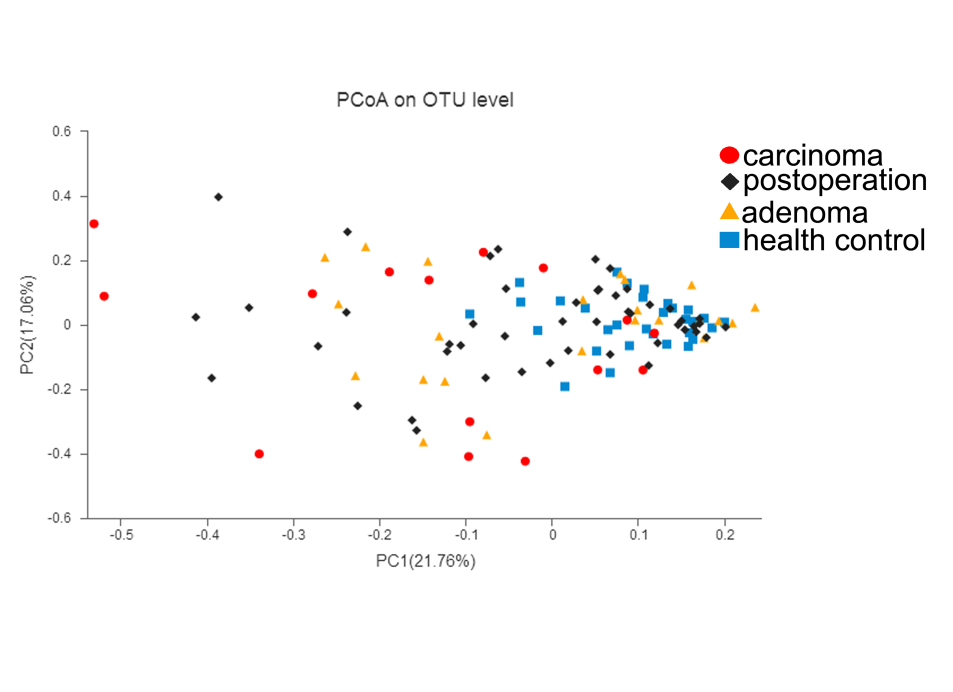


**Figure S4.** Principal-coordinated analysis based on Weighted Unifrac of healthy control, [adenoma](javascript:void(0);), carcinoma and postoperative patients.


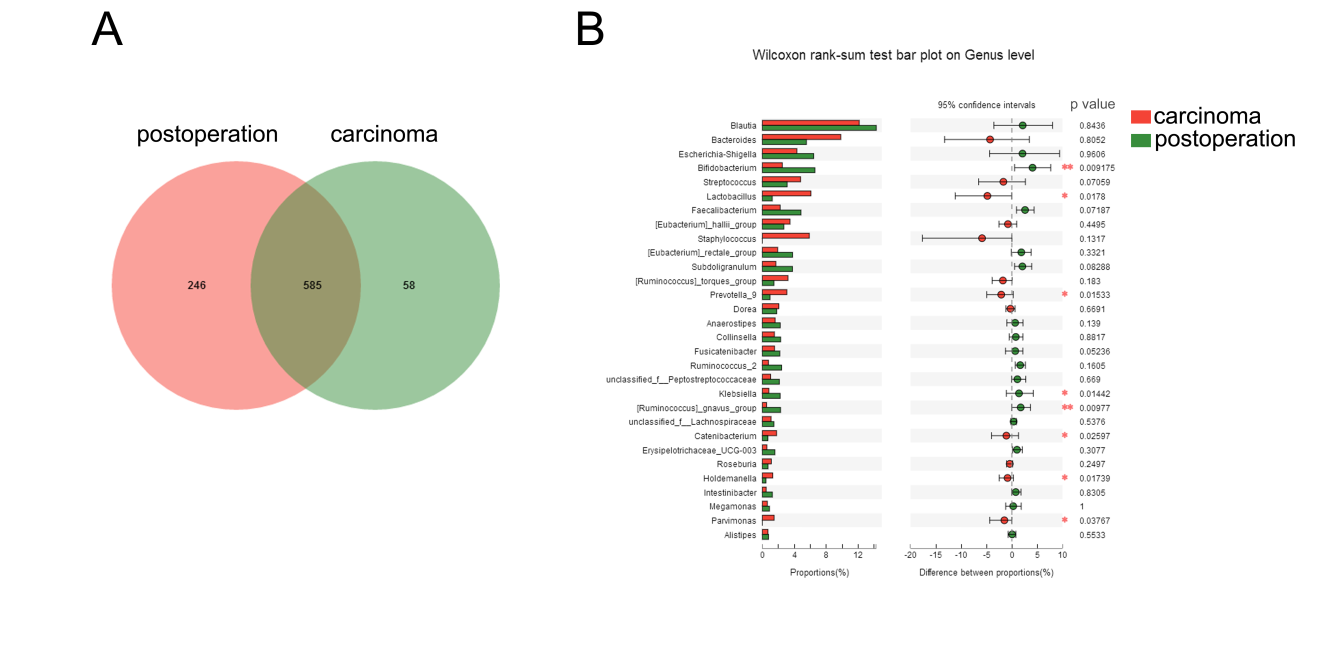


**Figure S5.** The difference of microbiota between carcinoma patients and postoperative patients in genus level. (A) Venn diagram showing the common genus between groups. (B) The relative abundance of genus in two groups. Wilcoxon rank-sum test, *p<0.05, **p<0.01.


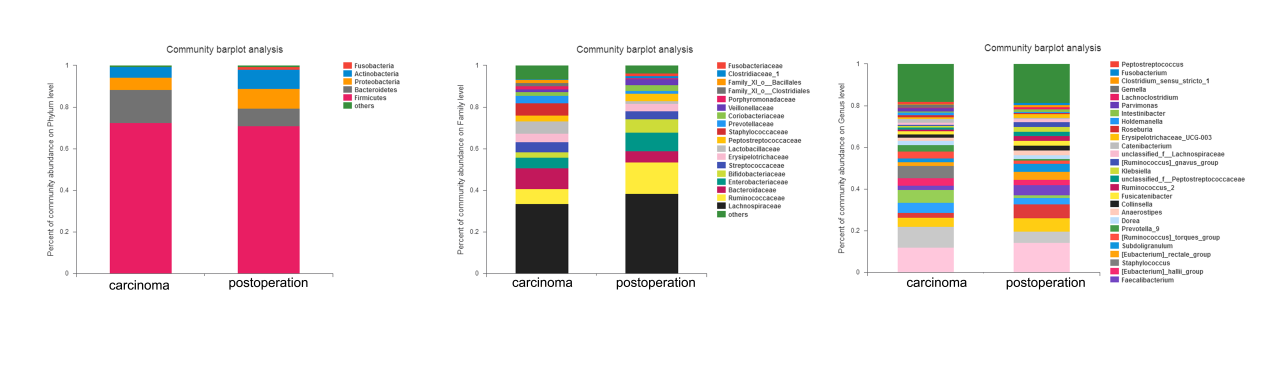


**Figure S6.** The relative abundance of faecal bacterial phyla, family and genus were clustered between carcinoma patients and postoperative patients. All OTUs with lower abundances were grouped as “others”.


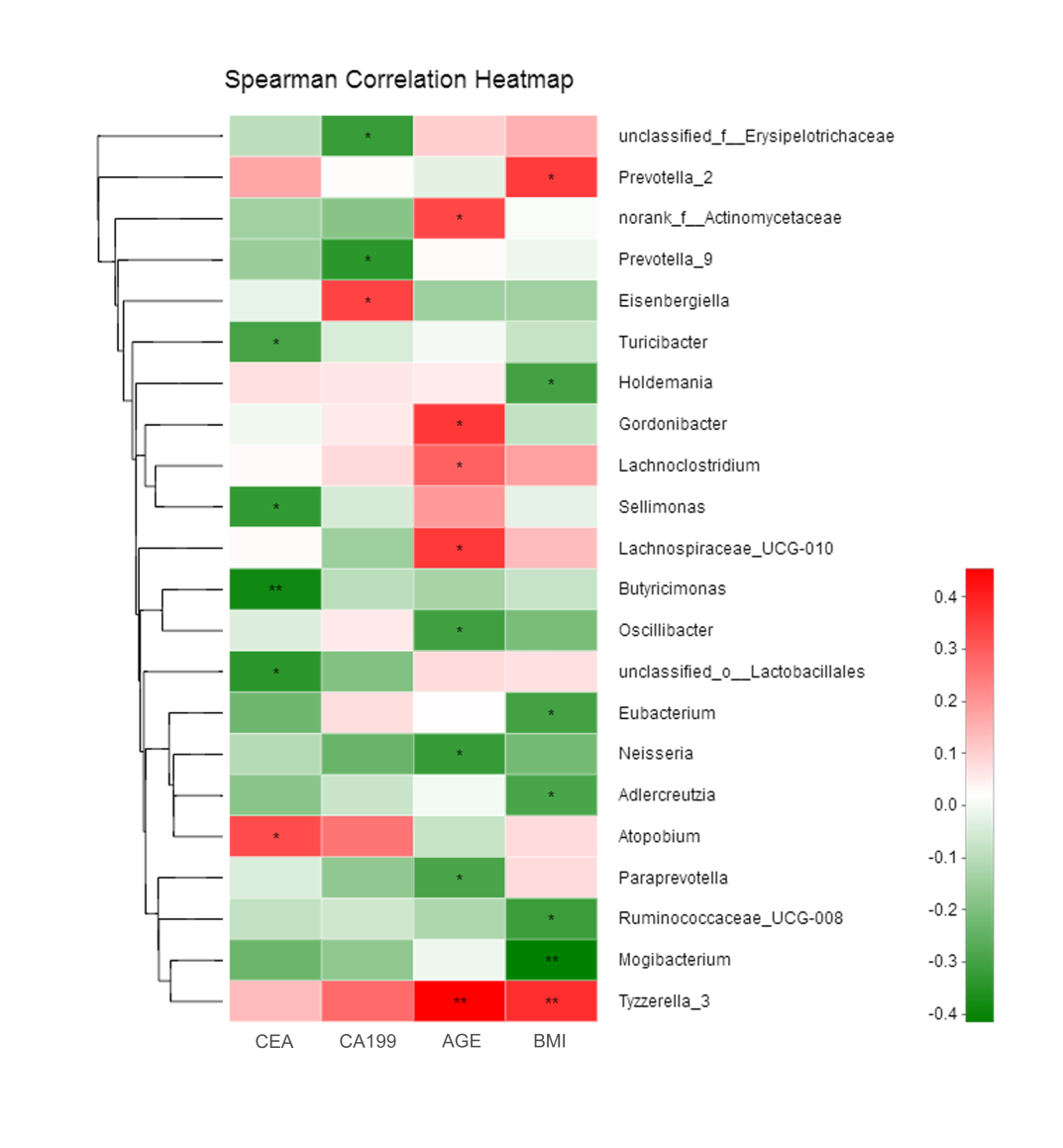


**Figure S7.** Colour-coded heatmap displaying the relationship among microbiota, CEA, CA19-9, age and BMI. The colour scale represents the correlation coefficient of bacteria and clinical index, with red and green indicating a positive and negative correlation, respectively. *p<0.05, **p<0.01. BMI, body mass index; CA19-9, carbohydrate antigen 19-9; CEA, carcino embryonic antigen.
