# Supplementary figures and images for: Gut microbiota in patients after surgical treatment for colorectal cancer
Source: Environ Microbiol. 2018 Dec 19;21(2):772–83. doi: 10.1111/1462-2920.14498 (PMC7379540; doi:10.1111/1462-2920.14498)

Receiver operating characteristic of selected genus

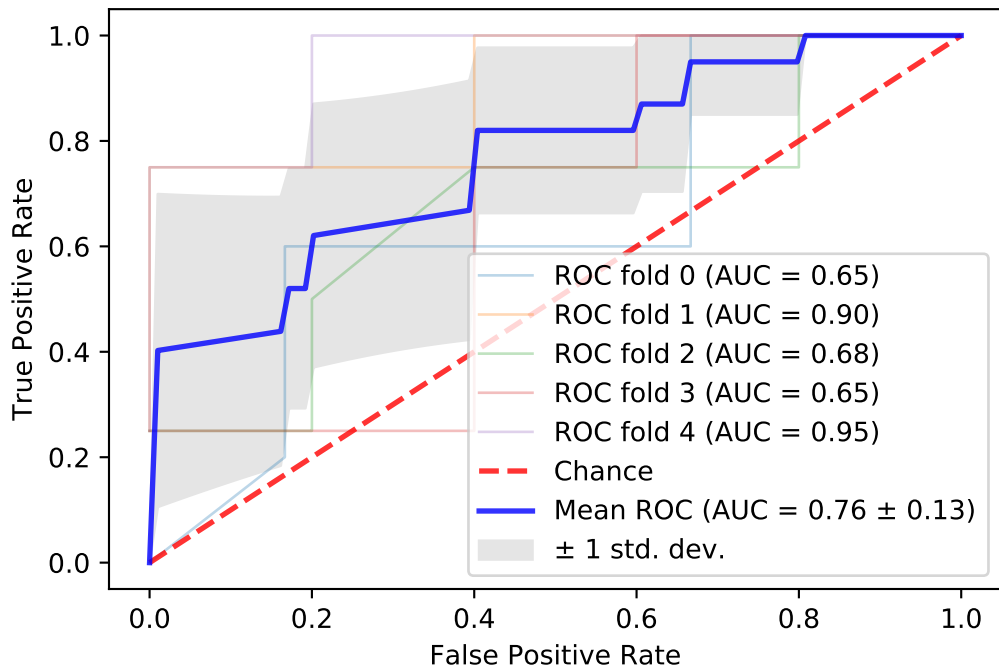

Supplement: Supplementary file 2 — Fig. S8. Receiver operating characteristic of selected genus [file EMI-21-772-s009.pdf]
