# Supplementary material for: Gut microbiota in patients after surgical treatment for colorectal cancer
Source: Environ Microbiol. 2018 Dec 19;21(2):772–83. doi: 10.1111/1462-2920.14498 (PMC7379540; doi:10.1111/1462-2920.14498)
Supplement: Supplementary file 4 — Table S2. The relative abundance of microbiota in genus level between carcinoma patients and postoperative patients. [file EMI-21-772-s002.docx]

**Table S2.** The relative abundance of microbiota in genus level between carcinoma patients and postoperative patients.

| Species name | carcinoma | | postoperation | |  |  |
| --- | --- | --- | --- | --- | --- | --- |
|  | Mean | SD | Mean | SD | P | Q |
| *Bifidobacterium* | 2.51E+00 | 4.89E+00 | 6.58E+00 | 9.58E+00 | 9.18E-03 | 2.01E-01 |
| *Lactobacillus* | 6.08E+00 | 1.14E+01 | 1.25E+00 | 3.59E+00 | 1.78E-02 | 2.17E-01 |
| *Prevotella 9* | 3.07E+00 | 5.41E+00 | 9.72E-01 | 2.53E+00 | 1.53E-02 | 2.17E-01 |
| *Klebsiella* | 8.21E-01 | 2.79E+00 | 2.23E+00 | 7.58E+00 | 1.44E-02 | 2.17E-01 |
| *Ruminococcus gnavus group* | 5.40E-01 | 2.03E+00 | 2.28E+00 | 5.39E+00 | 9.77E-03 | 2.01E-01 |
| *Catenibacterium* | 1.78E+00 | 5.32E+00 | 6.97E-01 | 4.31E+00 | 2.60E-02 | 2.78E-01 |
| *Holdemanella* | 1.31E+00 | 2.86E+00 | 4.62E-01 | 1.32E+00 | 1.74E-02 | 2.17E-01 |
| *Parvimonas* | 1.48E+00 | 5.45E+00 | 1.69E-03 | 4.51E-03 | 3.77E-02 | 3.48E-01 |
| *Gemella* | 1.40E+00 | 4.05E+00 | 1.43E-02 | 3.74E-02 | 1.13E-03 | 6.65E-02 |
| *Peptostreptococcus* | 1.10E+00 | 3.73E+00 | 5.37E-03 | 1.52E-02 | 3.17E-02 | 3.03E-01 |
| *Porphyromonas* | 8.63E-01 | 2.36E+00 | 6.96E-04 | 2.92E-03 | 5.44E-03 | 1.82E-01 |
| *Weissella* | 6.66E-01 | 2.58E+00 | 4.09E-02 | 1.56E-01 | 4.73E-02 | 3.62E-01 |
| *Granulicatella* | 6.56E-01 | 1.65E+00 | 2.02E-02 | 6.04E-02 | 6.99E-03 | 1.88E-01 |
| *Actinomyces* | 4.54E-01 | 1.26E+00 | 6.12E-02 | 9.33E-02 | 4.80E-02 | 3.62E-01 |
| *Prevotella 2* | 6.32E-02 | 1.22E-01 | 4.29E-01 | 2.72E+00 | 1.24E-02 | 2.10E-01 |
| *Unclassified Lactobacillales* | 2.48E-01 | 6.31E-01 | 1.13E-01 | 5.30E-01 | 4.05E-03 | 1.55E-01 |
| *Tyzzerella 3* | 9.65E-03 | 3.26E-02 | 2.39E-01 | 3.33E-01 | 1.34E-04 | 1.80E-02 |
| *Lachnospira* | 2.96E-02 | 6.52E-02 | 1.16E-01 | 2.93E-01 | 2.53E-02 | 2.78E-01 |
| *Corynebacterium 1* | 8.81E-02 | 2.86E-01 | 4.97E-04 | 2.79E-03 | 4.92E-02 | 3.62E-01 |
| *Sutterella* | 1.56E-03 | 3.81E-03 | 6.95E-02 | 2.20E-01 | 8.23E-03 | 2.01E-01 |
| *Faecalitalea* | 4.36E-03 | 1.10E-02 | 6.09E-02 | 1.90E-01 | 3.14E-02 | 3.03E-01 |
| *Eubacterium brachy group* | 4.05E-03 | 7.25E-03 | 3.94E-02 | 9.87E-02 | 2.75E-02 | 2.83E-01 |
| *Unclassified Clostridiales* | 3.11E-03 | 5.20E-03 | 2.15E-02 | 3.03E-02 | 1.65E-02 | 2.17E-01 |
| *Alloprevotella* | 2.24E-02 | 7.66E-02 | 1.59E-03 | 8.89E-03 | 1.57E-02 | 2.17E-01 |
| *Unclassified Oxalobacteraceae* | 1.87E-02 | 5.06E-02 | 2.48E-03 | 5.58E-03 | 1.24E-03 | 6.65E-02 |
| *Pseudomonas* | 1.96E-02 | 5.84E-02 | 5.96E-04 | 2.86E-03 | 6.71E-03 | 1.88E-01 |
| *Howardella* | 1.21E-02 | 1.84E-02 | 3.18E-03 | 1.39E-02 | 6.05E-05 | 1.62E-02 |
| *Atopobium* | 7.47E-03 | 1.03E-02 | 2.78E-03 | 7.52E-03 | 4.40E-02 | 3.62E-01 |
| *Ralstonia* | 4.67E-03 | 8.83E-03 | 8.94E-04 | 2.51E-03 | 1.88E-02 | 2.18E-01 |
| *Lawsonella* | 5.29E-03 | 1.80E-02 | 0.00E+00 | 0.00E+00 | 1.97E-03 | 8.81E-02 |
| *Microbacterium* | 2.18E-03 | 7.25E-03 | 0.00E+00 | 0.00E+00 | 1.25E-02 | 2.10E-01 |
| *Finegoldia* | 1.56E-03 | 2.28E-03 | 5.96E-04 | 1.85E-03 | 4.73E-02 | 3.62E-01 |
| *Parascardovia* | 1.25E-03 | 2.14E-03 | 0.00E+00 | 0.00E+00 | 3.05E-04 | 2.72E-02 |
| *Leptotrichia* | 1.25E-03 | 3.29E-03 | 0.00E+00 | 0.00E+00 | 1.25E-02 | 2.10E-01 |

Wilcoxon rank-sum test.
