# Supplementary material for: Gut microbiota in patients after surgical treatment for colorectal cancer
Source: Environ Microbiol. 2018 Dec 19;21(2):772–83. doi: 10.1111/1462-2920.14498 (PMC7379540; doi:10.1111/1462-2920.14498)
Supplement: Supplementary file 5 — Table S3. The average relative abundance of microbiota for five groups in heatmap. [file EMI-21-772-s003.docx]

| Genus | HC | CIT | adenoma | NDA | carcinoma |
| --- | --- | --- | --- | --- | --- |
| *Clostridium sensu stricto 1* | -2.385 | -2.576 | -2.880 | -3.245 | -3.264 |
| *Faecalibacterium* | -1.374 | -1.841 | -1.394 | -1.814 | -2.224 |
| *Ruminococcus gnavus group* | -3.523 | -3.608 | -4.086 | -3.138 | -4.795 |
| *Intestinibacter* | -2.318 | -2.975 | -3.008 | -3.525 | -3.051 |
| *Anaerostipes* | -1.697 | -2.030 | -1.882 | -2.292 | -2.713 |
| *Ruminococcus 2* | -2.161 | -2.429 | -2.611 | -3.739 | -3.212 |
| *Fusicatenibacter* | -1.732 | -2.230 | -1.902 | -2.432 | -2.744 |
| *Erysipelotrichaceae UCG 003* | -2.155 | -2.703 | -2.112 | -2.900 | -2.969 |
| *Bifidobacterium* | -1.970 | -1.855 | -2.456 | -1.689 | -2.887 |
| *Eubacterium hallii group* | -1.373 | -2.103 | -1.700 | -2.260 | -1.978 |
| *Ruminococcaceae UCG 013* | -2.859 | -2.850 | -3.020 | -3.364 | -3.369 |
| *Eubacterium coprostanoligenes group* | -2.594 | -2.732 | -2.870 | -3.466 | -3.269 |
| *unclassified Peptostreptococcaceae* | -1.775 | -2.109 | -2.195 | -2.749 | -2.340 |
| *unclassified Lachnospiraceae* | -1.762 | -2.165 | -1.818 | -2.197 | -2.089 |
| *Dorea* | -1.566 | -2.114 | -1.646 | -2.254 | -2.028 |
| *Klebsiella* | -3.509 | -3.293 | -3.845 | -3.457 | -4.545 |
| *Blautia* | -0.878 | -1.115 | -0.913 | -1.081 | -1.196 |
| *Catenibacterium* | -5.594 | -5.583 | -5.439 | -5.625 | -4.850 |
| *Haemophilus* | -4.611 | -4.517 | -4.785 | -4.921 | -4.497 |
| *Staphylococcus* | -5.517 | -5.472 | -5.688 | -5.244 | -5.492 |
| *Megamonas* | -5.267 | -5.010 | -4.816 | -5.031 | -5.024 |
| *Collinsella* | -2.988 | -2.896 | -2.332 | -3.168 | -2.414 |
| *Eubacterium rectale group* | -1.568 | -2.139 | -1.551 | -2.240 | -2.351 |
| *Lactobacillus* | -3.659 | -4.050 | -3.950 | -3.640 | -2.641 |
| *Coprococcus 2* | -4.214 | -3.808 | -3.607 | -4.861 | -3.769 |
| *Roseburia* | -1.923 | -2.433 | -2.333 | -3.074 | -2.372 |
| *Alistipes* | -3.305 | -2.962 | -3.650 | -3.890 | -3.049 |
| *Subdoligranulum* | -1.807 | -2.056 | -1.798 | -1.977 | -2.227 |
| *Dialister* | -4.254 | -3.705 | -4.109 | -4.358 | -4.281 |
| *Holdemanella* | -4.300 | -5.140 | -4.682 | -4.937 | -3.812 |
| *Coprococcus 3* | -2.477 | -3.100 | -2.598 | -3.405 | -2.462 |
| *Streptococcus* | -2.401 | -2.412 | -2.440 | -2.118 | -1.838 |
| *Ruminococcus torques group* | -1.637 | -2.265 | -1.946 | -2.222 | -1.911 |
| *Prevotella 9* | -4.140 | -4.472 | -3.319 | -4.147 | -2.860 |
| *Bacteroides* | -1.797 | -1.974 | -1.918 | -1.591 | -1.889 |
| *Lachnoclostridium* | -2.670 | -2.693 | -2.850 | -2.375 | -2.910 |
| *Escherichia Shigella* | -2.714 | -2.987 | -2.639 | -2.451 | -2.581 |

**Table S3.** The average relative abundance of microbiota for five groups in heatmap.

Data were showed with lg. HC, health controlsNDA, newly developed adenoma; CIT, clean intestine
