# Supplementary material for: Gut microbiota in patients after surgical treatment for colorectal cancer
Source: Environ Microbiol. 2018 Dec 19;21(2):772–83. doi: 10.1111/1462-2920.14498 (PMC7379540; doi:10.1111/1462-2920.14498)
Supplement: Supplementary file 6 — Table S4. The statistical difference abundance of genera with average abundance levels >0.5% between carcinoma patients and healthy controls. [file EMI-21-772-s004.docx]

**Table S4.** The statistical difference abundance of genera with average abundance levels >0.5% between carcinoma patients and healthy controls.

| Species name | carcinoma | | healthy control | |  |  |
| --- | --- | --- | --- | --- | --- | --- |
|  | Mean | SD | Mean | SD | P | Q |
| *Faecalibacterium* | 2.60E+00 | 2.50E+00 | 8.54E+00 | 7.35E+00 | 2.90E-03 | 4.54E-02 |
| *Eubacterium rectale group* | 2.30E+00 | 3.30E+00 | 5.67E+00 | 5.63E+00 | 1.17E-02 | 6.11E-02 |
| *Lactobacillus* | 6.38E+00 | 1.14E+01 | 7.33E-01 | 3.43E+00 | 1.52E-02 | 6.11E-02 |
| *Dorea* | 2.55E+00 | 2.16E+00 | 4.41E+00 | 2.47E+00 | 1.33E-02 | 6.11E-02 |
| *Streptococcus* | 5.47E+00 | 8.38E+00 | 1.10E+00 | 1.49E+00 | 1.93E-02 | 6.11E-02 |
| *Anaerostipes* | 1.83E+00 | 3.00E+00 | 3.62E+00 | 3.37E+00 | 5.18E-03 | 4.86E-02 |
| *Fusicatenibacter* | 1.76E+00 | 3.99E+00 | 3.46E+00 | 3.50E+00 | 9.74E-04 | 4.54E-02 |
| *Ruminococcus 2* | 9.32E-01 | 1.41E+00 | 3.99E+00 | 3.78E+00 | 7.23E-03 | 4.86E-02 |
| *Prevotella 9* | 3.33E+00 | 5.71E+00 | 1.12E+00 | 2.67E+00 | 2.41E-02 | 7.07E-02 |
| *Unclassified Peptostreptococcaceae* | 1.21E+00 | 1.29E+00 | 3.09E+00 | 2.78E+00 | 1.82E-02 | 6.11E-02 |
| *Erysipelotrichaceae UCG-003* | 7.00E-01 | 8.61E-01 | 3.09E+00 | 3.82E+00 | 2.49E-03 | 4.54E-02 |
| *Ruminococcus gnavus group* | 7.07E-01 | 2.66E+00 | 1.52E+00 | 3.43E+00 | 1.21E-02 | 6.11E-02 |
| *Clostridium sensu stricto 1* | 2.50E-01 | 4.00E-01 | 1.87E+00 | 3.23E+00 | 6.37E-03 | 4.86E-02 |
| *Unclassified Lachnospiraceae* | 5.62E-01 | 6.94E-01 | 1.25E+00 | 1.15E+00 | 5.96E-03 | 4.86E-02 |
| *Klebsiella* | 9.32E-01 | 3.20E+00 | 4.43E-01 | 1.33E+00 | 3.56E-02 | 9.84E-02 |

Wilcoxon rank-sum test.
