# Supplementary material for: Gut microbiota in patients after surgical treatment for colorectal cancer
Source: Environ Microbiol. 2018 Dec 19;21(2):772–83. doi: 10.1111/1462-2920.14498 (PMC7379540; doi:10.1111/1462-2920.14498)
Supplement: Supplementary file 7 — Table S5. The relationship among microbiota, CEA, CA19‐9, age and BMI. [file EMI-21-772-s005.docx]

**Table S5.** The relationship among microbiota, CEA, CA19-9, age and BMI.

| Species name | CEA | | CA199 | | AGE | | BMI | |
| --- | --- | --- | --- | --- | --- | --- | --- | --- |
|  | P | R | P | R | P | R | P | R |
| *Unclassified Erysipelotrichaceae* | 0.533 | -0.09 | 0.029 | -0.32 | 0.498 | 0.101 | 0.301 | 0.154 |
| *Prevotella 2* | 0.253 | 0.17 | 0.868 | 0.025 | 0.862 | -0.03 | 0.015 | 0.354 |
| *Norank Actinomycetaceae* | 0.346 | -0.14 | 0.215 | -0.18 | 0.022 | 0.333 | 0.952 | 0.009 |
| *Prevotella 9* | 0.309 | -0.15 | 0.019 | -0.34 | 0.861 | 0.026 | 0.95 | -0.01 |
| *Eisenbergiella* | 0.899 | -0.02 | 0.02 | 0.339 | 0.32 | -0.15 | 0.344 | -0.14 |
| *Turicibacter* | 0.042 | -0.3 | 0.752 | -0.05 | 1 | 0 | 0.609 | -0.08 |
| *Holdemania* | 0.626 | 0.073 | 0.679 | 0.062 | 0.716 | 0.054 | 0.04 | -0.3 |
| *Gordonibacter* | 0.968 | -0.01 | 0.712 | 0.055 | 0.013 | 0.361 | 0.584 | -0.08 |
| *Lachnoclostridium* | 0.848 | 0.029 | 0.585 | 0.082 | 0.049 | 0.289 | 0.229 | 0.179 |
| *Sellimonas* | 0.024 | -0.33 | 0.736 | -0.05 | 0.195 | 0.192 | 0.871 | -0.02 |
| *Lachnospiraceae UCG-010* | 0.864 | 0.026 | 0.319 | -0.15 | 0.013 | 0.359 | 0.374 | 0.133 |
| *Butyricimonas* | 0.007 | -0.39 | 0.521 | -0.1 | 0.382 | -0.13 | 0.614 | -0.08 |
| *Oscillibacter* | 0.802 | -0.04 | 0.704 | 0.057 | 0.036 | -0.31 | 0.155 | -0.21 |
| *Unclassified Lactobacillales* | 0.019 | -0.34 | 0.198 | -0.19 | 0.587 | 0.081 | 0.625 | 0.073 |
| *Eubacterium* | 0.127 | -0.23 | 0.615 | 0.075 | 0.877 | 0.023 | 0.04 | -0.3 |
| *Neisseria* | 0.479 | -0.11 | 0.11 | -0.24 | 0.028 | -0.32 | 0.14 | -0.22 |
| *Adlercreutzia* | 0.224 | -0.18 | 0.642 | -0.07 | 0.993 | -0 | 0.048 | -0.29 |
| *Atopobium* | 0.026 | 0.326 | 0.081 | 0.257 | 0.609 | -0.08 | 0.589 | 0.081 |
| *Paraprevotella* | 0.787 | -0.04 | 0.259 | -0.17 | 0.049 | -0.29 | 0.6 | 0.079 |
| *Ruminococcaceae UCG-008* | 0.574 | -0.08 | 0.677 | -0.06 | 0.427 | -0.12 | 0.031 | -0.32 |
| *Mogibacterium* | 0.115 | -0.23 | 0.248 | -0.17 | 0.949 | -0.01 | 0.004 | -0.42 |
| *Tyzzerella 3* | 0.362 | 0.136 | 0.063 | 0.273 | 0.001 | 0.454 | 0.009 | 0.378 |

Spearman rank correlation. BMI, body mass index; CA19-9, carbohydrate antigen 19-9; CEA, carcino embryonic antigen.
