# Supplementary material for: Gut microbiota in patients after surgical treatment for colorectal cancer
Source: Environ Microbiol. 2018 Dec 19;21(2):772–83. doi: 10.1111/1462-2920.14498 (PMC7379540; doi:10.1111/1462-2920.14498)
Supplement: Supplementary file 8 — Table S6. The LEfSe result of NDA and CIT in postoperative patients. [file EMI-21-772-s006.docx]

| Species name | Mean | LDA_value | Pvalue |
| --- | --- | --- | --- |
| Unclassified Peptostreptococcaceae | 4.507 | 4.130 | 0.020 |
| Ruminococcus 2 | 4.522 | 4.039 | 0.010 |
| Clostridium sensu stricto 1 | 4.205 | 3.790 | 0.038 |
| Coprococcus 2 | 4.093 | 3.630 | 0.039 |
| Finegoldia | 1.033 | 3.453 | 0.036 |
| Eubacterium coprostanoligenes group | 3.918 | 3.250 | 0.044 |
| Ruminococcaceae UCG 002 | 3.651 | 3.182 | 0.005 |
| Abiotrophia | 1.192 | 3.034 | 0.049 |
| Norank Mollicutes RF9 | 3.455 | 2.962 | 0.005 |
| norank Christensenellaceae | 1.773 | 2.907 | 0.049 |
| Lachnospiraceae NK4A136 group | 3.381 | 2.900 | 0.047 |
| Hydrogenoanaerobacterium | 1.368 | 2.898 | 0.020 |
| Ruminococcaceae NK4A214 group | 3.337 | 2.870 | 0.046 |
| Ruminococcaceae UCG 005 | 3.361 | 2.869 | 0.025 |
| Moryella | 2.003 | 2.833 | 0.012 |
| Atopobium | 1.669 | 2.822 | 0.020 |
| Morganella | 3.006 | 2.751 | 0.049 |
| Prevotella 7 | 2.680 | 2.726 | 0.022 |
| Oscillibacter | 1.760 | 2.682 | 0.039 |
| Unclassified oriobacteriaceae | 1.823 | 2.672 | 0.006 |
| norank Coriobacteriaceae | 3.170 | 2.665 | 0.046 |
| Desulfovibrio | 3.176 | 2.654 | 0.043 |
| Anaerotruncus | 3.032 | 2.642 | 0.014 |
| Ruminococcaceae UCG 010 | 2.852 | 2.601 | 0.036 |
| Butyricimonas | 2.708 | 2.562 | 0.028 |
| Solobacterium | 1.866 | 2.535 | 0.015 |

Table S6. the LEfSe result of NDA and CIT in postoperative patients.

Kruskal–Wallis rank-sum test. NDA, newly developed adenoma; CIT, clean intestine
